# Prediction of HIV-1 and HIV-2 proteins by using Chou’s pseudo amino acid compositions and different classifiers

**DOI:** 10.1038/s41598-018-20819-x

**Published:** 2018-02-05

**Authors:** Juan Mei, Ji Zhao

**Affiliations:** School of Internet of Things Engineering, Wuxi City College of Vocational Technology, Wuxi, 214153 China

## Abstract

Human immunodeficiency virus (HIV) is the retroviral agent that causes acquired immune deficiency syndrome (AIDS). The number of HIV caused deaths was about 4 million in 2016 alone; it was estimated that about 33 million to 46 million people worldwide living with HIV. The HIV disease is especially harmful because the progressive destruction of the immune system prevents the ability of forming specific antibodies and to maintain an efficacious killer T cell activity. Successful prediction of HIV protein has important significance for the biological and pharmacological functions. In this study, based on the concept of Chou’s pseudo amino acid (PseAA) composition and increment of diversity (ID), support vector machine (SVM), logisitic regression (LR), and multilayer perceptron (MP) were presented to predict HIV-1 proteins and HIV-2 proteins. The results of the jackknife test indicated that the highest prediction accuracy and CC values were obtained by the SVM and MP were 0.9909 and 0.9763, respectively, indicating that the classifiers presented in this study were suitable for predicting two groups of HIV proteins.

## Introduction

Human immunodeficiency virus (HIV) is a retrovirus of the lentivirus family; it is thought to have originated in non-human primates in sub-Saharan Africa and transferred to humans in the 20th century^1–4^. There are two types of human immunodeficiency viruses: HIV-1 and HIV-2. The epidemiological and biological characteristics of HIV-1 and HIV-2 exhibit major differences, whereas HIV-2 is confined mainly to West Africa in only a minority of infected individuals, HIV-1 is spread globally. The proteins encoded by the HIV genome contains genes are defined as HIV proteins. Three major genes are contained in the HIV genome; the major structural proteins as well as essential enzymes are encoded by them. Until now, 381 HIV-1 proteins and 109 HIV-2 proteins are contained in the Swiss-Prot database^5^, respectively. HIV-1 and HIV-2 are two different types of the HIV. Because of this, HIV-2 is a closely related retrovirus of HIV-1, but the difference still exists as well. The difference of HIV-1 proteins and HIV-2 proteins is that the vpx proteins found in HIV-2 are replaced by the vpu proteins in HIV-1^6^. In addition, the protease enzymes from the two retroviruses share about 50% sequence identity. Both HIV-1 and HIV-2 cause AIDS in humans^7–10^. HIV infects cells of the immune systems; such infection is characterized by the gradual loss of the CD4^+^ T cells and a progressive immune deficiency that leads to opportunistic infections and ultimately death^11–13^. Since the identification of HIV over thirty years ago, sixty million people have been infected with HIV; nearly half of them have died. It has reduced life expectancy, slowed economic growth, and deepened household poverty. During the past two decades or so, the following two strategies have been often adopted to find drugs against AIDS (acquired immunodeficiency syndrome). One is to target the HIV (human immunodeficiency virus) reverse transcriptase^14–19^; the other is to design HIV protease inhibitors^20–24^.

With more and more people are infected by HIV, successful identification of HIV proteins may have important significance for global fight against HIV. Although, many efforts have been made to identification of HIV proteins by experimental methods, it is time consuming and costly. In recent years, several machine learning methods have been developed for predicting different groups of proteins by using sequence derived features, and good prediction results are obtained. So, the present work reported on the machine learning methods for prediction of the HIV-1 proteins and HIV-2 proteins, using the concept of Chou’s pseudo amino acid (PseAA) composition and increment of diversity (ID).

Computational algorithms, such as support vector machine (SVM)^25^ and increment of diversity (ID)^26^ have been developed in protein classification based on amino acid (AA) compositions and pseudo amino acid (PseAA) compositions^27,28^. Compared with the conventional amino acid (AA) composition, the pseudo amino acid (PseAA) composition can incorporate much more information of a protein sequence^27–31^.The pseudo amino acid (PseAA) composition can be considered as another simple representative form of protein’s neighborhood information. The increment of diversity (ID) is a measure of the whole uncertainly and total information of a system^26^. This algorithm has been used in the recognition of protein structural class^32^, the exon-intron splice site prediction^26^, and conotoxins superfamily prediction^33^ in recent years. However, until now, these are no algorithm for predicting HIV proteins. To fill this gap, in this study, the HIV-1 proteins and HIV-2 proteins were downloaded from the Swiss-Prot database^5^, and the amino acid (AA) compositions and pseudo amino acid (PseAA) compositions of HIV proteins were used as the input parameters of ID algorithm. Then, the HIV-1 proteins and HIV-2 proteins were predicted by the support vector machine (SVM)^25^, logisitic regression (LR), and multilayer perceptron (MP) by using the ID values as the input parameters. The jackknife test was used to evaluate the prediction quality of these algorithms, and good predictive results were obtained in this study, indicating that these algorithms were suitable for predicting HIV proteins. The efficiency in prediction of HIV proteins may facilitate the search for new diagnostic tools and drug targets of HIV. The findings presented in this study may provide some useful help for discovery of new biomarkers of HIV. To develop a really useful sequence-based statistical predictor for a biological system as reported in a series of recent publications^34–43^, one should observe the 5-step rule^29^; i.e., making the following five steps very clear: (i) how to construct or select a valid benchmark dataset to train and test the predictor; (ii) how to formulate the biological sequence samples with an effective mathematical expression that can truly reflect their intrinsic correlation with the target to be predicted; (iii) how to introduce or develop a powerful algorithm (or engine) to operate the prediction; (iv) how to properly perform cross-validation tests to objectively evaluate the anticipated accuracy of the predictor; (v) how to establish a user-friendly web-server for the predictor that is accessible to the public. Below, we are to describe how to deal with these steps one-by-one.

## Results

### Comparison on 20 amino acid compositions

**Figure 1:**
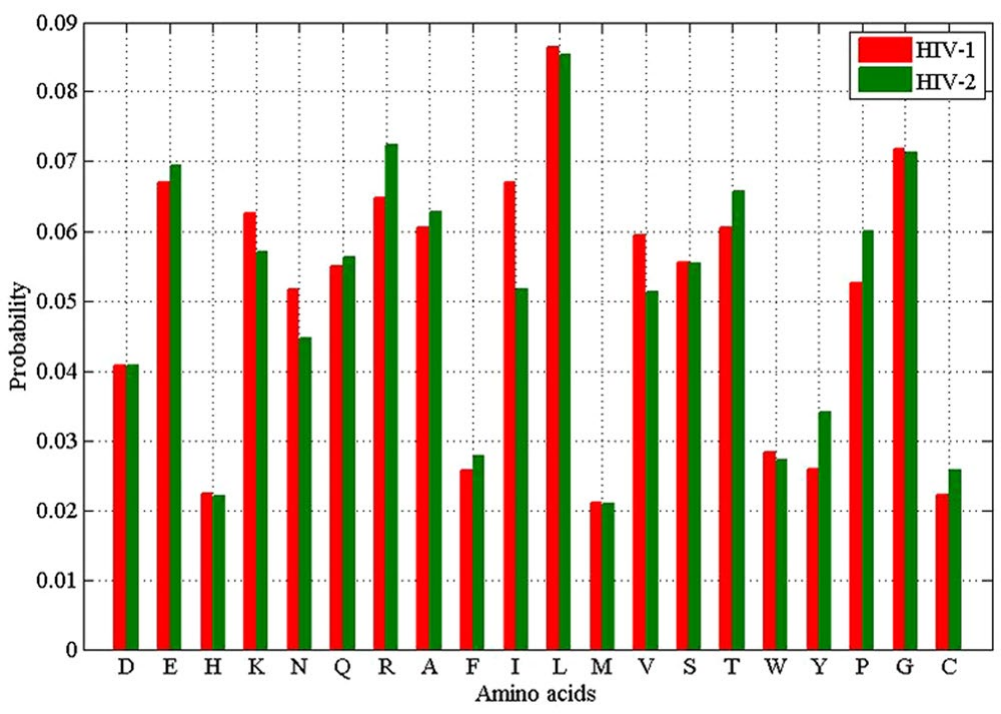
Amino acid frequencies of each amino acid in two HIV groups.

**Figure 2:**
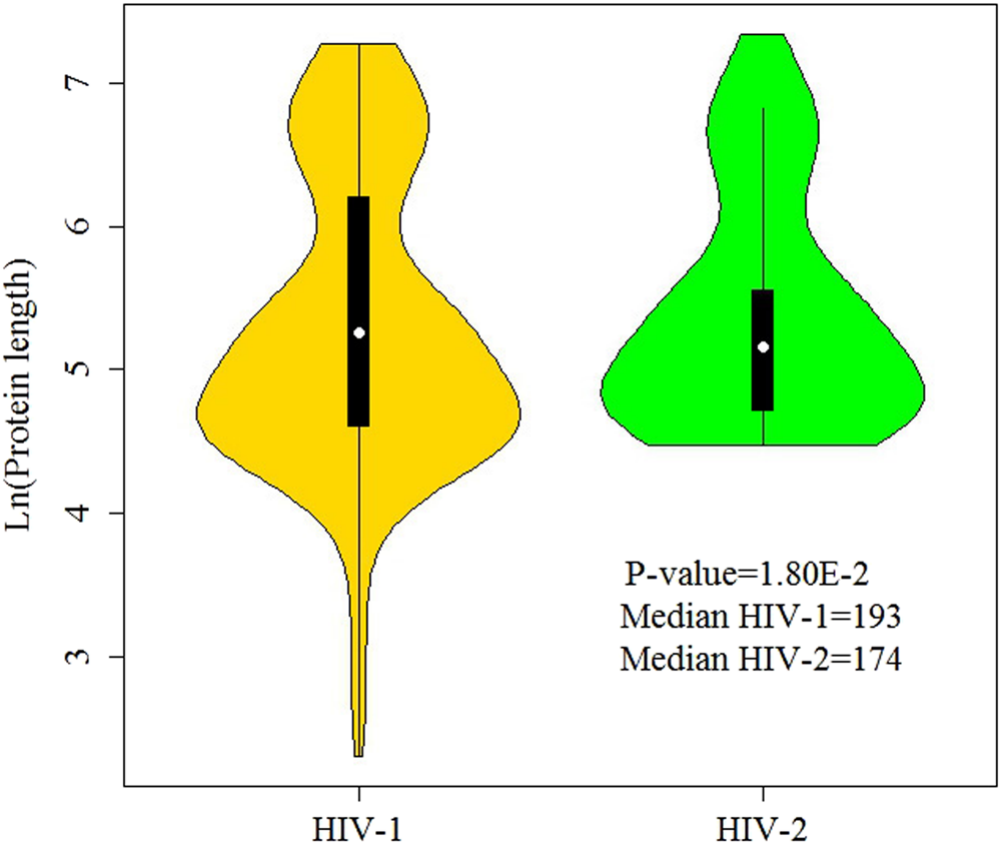
The violin plots for protein lengths of HIV-1 proteins and HIV-2 proteins.

The amino acid (AA) compositions of protein sequences have been widely used in classification of various groups of proteins in recent years^28,29,44–47^. Some studies indicated that the biological function of a protein was mainly dependent on its amino acid compositions. In this study, the overall frequencies of the 20 amino acids for 242 HIV-1 proteins and 86 HIV-2 proteins were plotted (Fig. 1). Figure 1 illustrated that the amino acids of Glu (E), Lys (K), Gln (Q), Arg (R), Ala (A), Ile (I), Leu (L), Val (V), Ser (S), Thr (T), Pro (P) and Gly (G) were preferred to have high frequencies (frequency > 5%) in both HIV-1 proteins and HIV-2 proteins. To further study the difference in amino acid usage, we compared the percentages of each amino acid, respectively, between the HIV-1 proteins and HIV-2 proteins (Table 1). The Wilcoxon tests revealed that Arg (R), Phe (F), Ile (I), Val (V), Thr (T), Tyr (Y) and Pro (P) had significant differences in the frequencies of amino acid usage. Among these amino acids, Arg (R), Ile (I), Val (V), Thr (T) and Pro (P) had high frequencies (frequency > 5%) for both HIV-1 proteins and HIV-2 proteins. In addition to the amino acid usage, the protein lengths of two protein groups were analyzed (Fig. 2). The median protein length of 242 HIV-1 proteins were longer than the median protein length of 86 HIV-2 proteins, and the difference between them was significant (193 versus 174, P-value = 1.80E-2; Wilcoxon test).

**Table 1:** The frequencies and P-values of 20 amino acids for 242 HIV-1 proteins and 86 HIV-2 proteins.

Amino acids	Abbreviations	Frequency		P-value
		HIV-1 proteins	HIV-2 proteins	
Asp	D	0.0407	0.0406	1.24E-01
Glu	E	0.067	0.0694	2.47E-01
His	H	0.0223	0.0219	2.03E-01
Lys	K	0.0625	0.057	1.46E-01
Asn	N	0.0516	0.0445	7.32E-02
Gln	Q	0.0549	0.0562	5.35E-01
Arg	R	0.0647	0.0723	3.49E-02
Ala	A	0.0605	0.0626	8.60E-02
Phe	F	0.0256	0.0276	7.71E-03
Ile	I	0.0669	0.0516	6.55E-04
Leu	L	0.0864	0.0853	4.41E-01
Met	M	0.0211	0.0208	7.61E-01
Val	V	0.0594	0.0512	2.15E-06
Ser	S	0.0555	0.0553	2.13E-01
Thr	T	0.0604	0.0656	8.19E-04
Trp	W	0.0283	0.0272	3.72E-01
Tyr	Y	0.0259	0.034	3.93E-04
Pro	P	0.0525	0.0599	1.05E-02
Gly	G	0.0718	0.0712	3.31E-01
Cys	C	0.0221	0.0257	6.33E-02

### F-scores of 20 amino acid compositions

**Figure 3:**
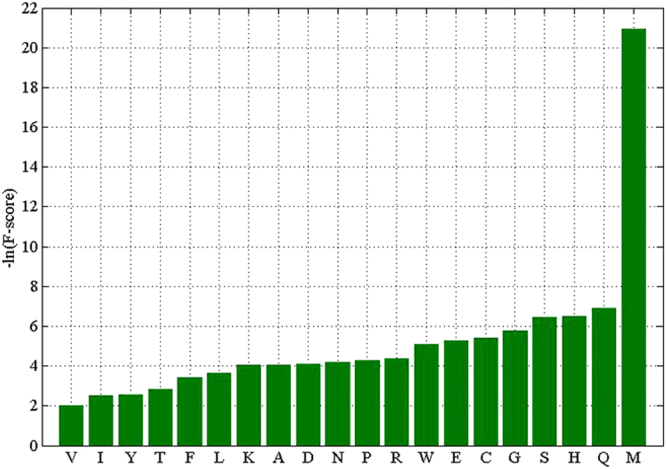
The F-scores of 20 amino acids. In this figure, x-axis represents the 20 amino acids, y-axis represents the-ln (F-score).

In this study, the F-scores of 20 amino acid compositions for HIV-1 proteins and HIV-2 proteins were also calculated for roughly evaluating the differences between amino acid compositions (Fig. 3). The larger the F-score was, the more likely this feature was more discriminative. As illustrated in Fig. 3, we found that Val (V) was the most discriminative feature, whereas Met (M) was the least discriminative feature, which confirmed the P-values of the Wilcoxon test for Val (V) and Met (M). We also found that most of the F-scores of 20 amino acids were low. The low F-scores of 20 amino acids were easy to understand, as most of the differences between HIV-1 proteins and HIV-2 proteins in amino acid usage were marginally or not significant. We hope that the F-scores of 20 amino acids illustrated in Fig. 3 may give us some quantitative indices for discriminating HIV-1 proteins and HIV-2 proteins. However, we should also keep in mind that the discrimination of each property was roughly estimated by the F-score, and further investigations will be required to prove the reliability and usefulness of this method.

### Prediction of HIV-1 proteins and HIV-2 proteins by the ID algorithm

In this study, the 20 amino acid compositions, 400 dipeptide compositions, 6 amino acid hydropathy compositions and 36 hydropathy dipeptide compositions were selected as the input parameters of the ID algorithm. The jackknife test was applied to examine the ID algorithm. The performances of ID algorithm for prediction of HIV-1 proteins and HIV-2 proteins were enumerated in Table 2. In this table, the best predictive results were obtained by selecting the 400 dipeptide compositions as the input parameters of the ID algorithm. For HIV-1 protein prediction, the results of jackknife test indicated that the sensitivity, specificity and CC value were 82.23%, 99.00% and 0.7215, respectively. For HIV-2 protein prediction, the results of jackknife test indicated that the sensitivity, specificity and CC value were 97.67%, 66.14% and 0.7215, respectively.

**Table 2:** The performance of ID algorithm for prediction of HIV-1 proteins and HIV-2 proteins.

Parameters	Types	Sn(%)	Sp(%)	Acc	CC
A_1_	HIV-1	43.39	95.45	0.5671	0.3500
	HIV-2	94.19	37.16	0.5671	0.3500
A_2_	HIV-1	82.23	99.00	0.8628	0.7215
	HIV-2	97.67	66.14	0.8628	0.7215
H_1_	HIV-1	55.79	88.82	0.6220	0.3177
	HIV-2	80.23	39.20	0.6220	0.3177
H_2_	HIV-1	64.46	95.71	0.7165	0.4955
	HIV-2	91.86	47.88	0.7165	0.4955

(A_1_: amino acid composition; A_2_: dipeptide composition; H_1_: amino acid hydropathy composition; H_2_: hydropathy dipeptide composition).

### Prediction of HIV-1 proteins and HIV-2 proteins by three different classifiers

In order to improve the prediction accuracy, the SVM, LR and MP were also applied to predict the HIV-1 proteins and HIV-2 proteins. In this study, the 20 amino acid compositions, 400 dipeptide compositions, 6 amino acid hydropathy compositions and 36 hydropathy dipeptide compositions were selected as the input parameters of the ID algorithm, and four kinds of ID values were calculated. Four kinds of ID values were combined and selected as the input parameters of SVM, LR and MP. All the predictive results were shown in Table 3. As shown in Table 3, the predictive results were improved by using the ID values as the input parameters of the SVM, LR and MP, when compared with the predictive results of the ID algorithm. Generally speaking, for HIV-1 protein and HIV-2 protein prediction, the better sensitivity, accuracy and CC value were obtained by the SVM, LR and MP.

**Table 3:** The performance of different classifiers for prediction of HIV-1 proteins and HIV-2 proteins.

Classifier	Parameters	HIV-1		HIV-2			
		Sn(%)	Sp(%)	Sn(%)	Sp(%)	Acc	CC
SVM	ID(A_1_) + ID(A_2_)	99.17	96.39	89.53	97.47	0.9665	0.9124
	ID(A_2_) + ID(H_1_)	100.00	97.58	93.02	100.00	0.9817	0.9527
	ID(A_2_) + ID(H_2_)	98.76	96.37	89.53	96.25	0.9634	0.9043
	ID(A_2_) + ID(A_1_) + ID(H_1_)	99.59	98.77	96.51	98.81	0.9878	0.9684
	ID(A_2_) + ID(A_1_) + ID(H_2_)	99.59	99.18	97.67	98.82	0.9909	0.9763
	ID(A_2_) + ID(H_1_) + ID(H_2_)	100.00	97.58	93.02	100.00	0.9817	0.9527
	ID(A_2_) + ID(A_1_) + ID(H_1_) + ID(H_2_)	99.59	98.37	95.35	98.8	0.9848	0.9604
Logisiticregression	ID(A_1_) + ID(A_2_)	98.76	97.55	93.02	96.39	0.9726	0.9285
	ID(A_2_) + ID(H_1_)	98.76	99.58	98.84	96.59	0.9878	0.9688
	ID(A_2_) + ID(H_2_)	98.35	97.54	93.02	95.24	0.9695	0.9207
	ID(A_2_) + ID(A_1_) + ID(H_1_)	98.76	99.17	97.67	96.55	0.9848	0.9608
	ID(A_2_) + ID(A_1_) + ID(H_2_)	97.52	97.93	94.19	93.1	0.9665	0.9137
	ID(A_2_) + ID(H_1_) + ID(H_2_)	98.76	99.58	98.84	96.59	0.9878	0.9688
	ID(A_2_) + ID(A_1_) + ID(H_1_) + ID(H_2_)	98.35	98.76	96.51	95.4	0.9787	0.9451
MultilayerPerceptron	ID(A_1_) + ID(A_2_)	97.93	97.53	93.02	94.12	0.9665	0.9130
	ID(A_2_) + ID(H_1_)	98.76	99.17	97.67	96.55	0.9848	0.9608
	ID(A_2_) + ID(H_2_)	98.35	97.54	93.02	95.24	0.9695	0.9207
	ID(A_2_) + ID(A_1_) + ID(H_1_)	98.76	98.76	96.51	96.51	0.9817	0.9527
	ID(A_2_) + ID(A_1_) + ID(H_2_)	99.17	96.77	91.01	97.59	0.9698	0.9225
	ID(A_2_) + ID(H_1_) + ID(H_2_)	99.59	99.18	97.67	98.82	0.9909	0.9763
	ID(A_2_) + ID(A_1_) + ID(H_1_) + ID(H_2_)	99.17	99.17	97.67	97.67	0.9878	0.9685

(A_1_: amino acid composition; A_2_: dipeptide composition; H_1_: amino acid hydropathy composition; H_2_: hydropathy dipeptide composition).

Based on the ID values, the 242 HIV-1 proteins and 86 HIV-2 proteins were predicted by the jackknife test. In the jackknife test, when using ID(A_2_), ID(A_1_) and ID(H_2_) as the input parameters of SVM for predicting the HIV-1 proteins and HIV-2 proteins, the overall accuracy of 0.9909 and the CC value of 0.9763 were obtained, which were the highest overall accuracy and CC value in this study. The same prediction results can also be obtained by using ID(A_2_), ID(H_1_) and ID(H_2_) as the input parameters of MP. In the jackknife test, the sensitivity (Sn) and specificity (Sp) were 99.59% and 99.18% for HIV-1 proteins, 97.67% and 98.82% for HIV-2 proteins by using ID(A_2_), ID(A_1_) and ID(H_2_) as the input parameters of SVM. All of the predictive results presented in Table 3 clearly indicated that the predictive successful rates of SVM, LR and MP were higher than those of the ID algorithm, and SVM, LR and MP were suitable for predicting two groups of HIV proteins.

## Discussion

The amino acid compositions of protein sequences have been widely used in classification of various groups of proteins in recent years. In this study, we used the amino acid compositions as the input parameters of increment of diversity (ID) to predict HIV-1 proteins and HIV-2 proteins. Before using these parameters, we wanted to show difference in the overall frequencies of the 20 amino acids for 242 HIV-1 proteins and 86 HIV-2 proteins. So, the frequencies and P-values of 20 amino acids for HIV-1 proteins and HIV-2 proteins were illustrated in Table 1.

In this study, the 20 amino acid compositions, 400 dipeptide compositions, 6 amino acid hydropathy compositions and 36 hydropathy dipeptide compositions were selected as the input parameters of the ID algorithm. Table 2 illustrated the sensitivity, specificity, accuracy, and correlation coefficient for predicting the HIV-1 proteins and HIV-2 proteins by the jackknife test. In this table, the readers can clearly found that the best prediction results were obtained by the 400 dipeptide compositions. So, in the next section, we combined the ID values of 400 dipeptide compositions with the ID values of three other compositions as the input parameters of SVM, LR and MP to predict two groups of HIV proteins.

As shown in some previous work for predicting the groups of proteins^27,32,48–52^, 20 amino acid compositions, 400 dipeptide compositions, 6 amino acid hydropathy compositions and 36 hydropathy dipeptide compositions were used as the input parameters. The prediction results of these work clearly indicated that better prediction quality was obtained by the 400 dipeptide compositions than three other parameters. Compared with 20 amino acid compositions which were the single wise amino acid compositions, the 400 dipeptide compositions took into account the sequence coupling effect^49^. More accurate correlation of the structure of a protein sequence was reflected in the 400 dipeptide compositions. So, the improved prediction quality can be obtained by the 400 dipeptide compositions. Compared with 6 amino acid hydropathy compositions and 36 hydropathy dipeptide compositions which only had 6 feature vectors and 36 feature vectors, more feature vectors were contained in the 400 dipeptide compositions. Thus, more information was contained in the 400 dipeptide compositions. This may be why the better prediction results could be obtained by 400 dipeptide compositions when compared with 6 amino acid hydropathy compositions and 36 hydropathy dipeptide compositions.

**Figure 4:**
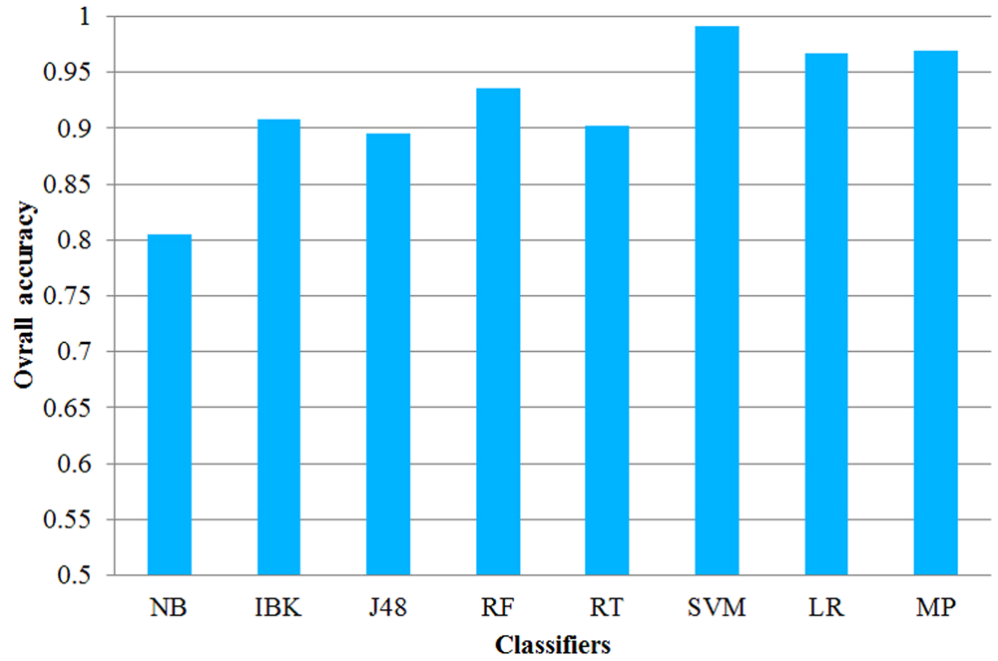
The comparison results of support vector machine (SVM), logisitic regression (LR), and multilayer perceptron (MP) with the naïve bayes (NB), IBK, J48, random forest (RF) and random tree (RT).

For comparing the prediction results of other machine learning algorithms with those of the SVM, LR and MP, the naïve bayes (NB), IBK, J48, random forest (RF) and random tree (RT) that were implemented in Weka (version 3.8.0) were used. The ID(A_2_), ID(A_1_) and ID(H_2_) were used as the input parameters of these machine learning algorithms for prediction the HIV-1 proteins and HIV-2 proteins. The performance of these classifiers for predicting two groups of HIV proteins was evaluated by the jackknife tests, and all the overall accuracies were shown in Fig. 4. As illustrated in this figure, we found that the overall accuracies of the SVM, LR and MP were higher than those of the NB, IBK, J48, RF and RT. Based on this, we can conclude that the SVM, LR and MP may be more suitable for predicting HIV-1 proteins and HIV-2 proteins.

The successful prediction of HIV-1 proteins and HIV-2 proteins indicated that the algorithms presented in this study were promising approaches. The experience gained from the above example indicated that the 400 dipeptide compositions and increment of diversity (ID) were suitable for predicting the HIV-1 proteins and HIV-2 proteins. The 400 dipeptide compositions may be used to improve the prediction quality; these predictive results were significant higher than the predictive results obtained by other parameters. It was also evidence that the primary sequences contained important information determined protein advance structure. In addition, we found that when using the ID values as the parameters of SVM, LR and MP can reduce dimension of input vectors, improving calculating efficiency and extract important classify information. We hope these algorithms will be helpful for identification of HIV proteins in the future.

In 2017, Behbahani *et al*.^53^. published the work for discrimination of HIV-1 and HIV-2 proteins. There were some differences between our work and the work of Behbahani *et al*. First, in the work of Behbahani *et al*., the protein sequences of HIV-1 and HIV-2 proteins were downloaded from the NCBI, the sequence identity was analyzed by the CD-HIT program, and sequence identity cutoff used in this study was 95%. The numbers of HIV-1 and HIV-2 protein sequences were 21 and 16, respectively. Compared with the work of Behbahani *et al*., our work used the different dataset, different sequence identity culling cutoff, and the numbers of HIV-1 and HIV-2 proteins were more than the work of Behbahani *et al*. Second, the work of Behbahani *et al*. focused on comparing HIV-1 and HIV-2 by using statistical analysis. They compared the difference in HIV-1 and HIV-2 by pseudo amino acid composition, conventional amino acid composition, physicochemical properties, secondary structures and structural motifs. Support vector machine algorithm was used for comparison of two protein groups. Only a little work was on the prediction of HIV-1 and HIV-2 proteins. However, in our work, we focused on prediction of HIV-1 and HIV-2 proteins by different classifier, and tried many methods to improve the prediction results. Although, we have compared the difference on 20 amino acid compositions between HIV-1 and HIV-2 proteins, the method was different with the work of Behbahani *et al*. We used the Wilcoxon tests and F-scores to study the difference between amino acid compositions in HIV-1 and HIV-2 proteins.

With the explosive growth of biological sequences in the post-genomic era, one of the most important but also most difficult problems in computational biology is how to express a biological sequence with a discrete model or a vector, yet still keep considerable sequence-order information or key pattern characteristic. This is because all the existing machine-learning algorithms can only handle vector but not sequence samples. However, a vector defined in a discrete model may completely lose all the sequence-pattern information. To avoid completely losing the sequence-pattern information for proteins, the pseudo amino acid composition^54,55^ was proposed. Ever since the concept of PseAAC was proposed, it has been widely used in nearly all the areas of computational proteomics^56–60^. Encouraged by the successes of using PseAAC to deal with protein/peptide sequences, the concept of PseKNC (Pseydo K-tuple Nucleotide Composition)^61^ was developed for generating various feature vectors for DNA/RNA sequences and it has been found very useful in genome analysis as well^34,62^. Particularly, recently a very powerful web-server called ‘Pse-in-One’^63^ and its updated version ‘Pse-in-One2.0’^64^ have been established that can be used to generate any desired feature vectors for protein/peptide and DNA/RNA sequences according to the need of users’ studies. As pointed out in the work of Chou and Shen^65^ and demonstrated in a series of recent publications^34–43,66^, user-friendly and publicly accessible web-servers represent the future direction for developing practically more useful prediction methods and computational tools. Actually, many practically useful web-servers have increasing the impacts of the relevant methods on medical science^56^, driving medicinal chemistry into an unprecedented revolution^56^, we shall make efforts in our future work to provide a web-server for the prediction method presented in this paper.

## Materials and Methods

### The HIV protein dataset

The dataset was downloaded from the Swiss-Prot (version 57.0) (http://www.uniprot.org/)^5^. This dataset contained 381 HIV-1 protein sequences and 109 HIV-2 protein sequences. The sequence identity was analyzed by a culling program PISCES (http://dunbrack.fccc.edu/PISCES.php)^67,68^. The distribution of their sequence identity percentage was shown in Table 4. In order to get enough number of protein sequences, HIV-1 dataset and HIV-2 dataset with ≤90% identity were used. The redundant protein sequences with more than 90% identity were deleted by a culling program: PISCES (http://dunbrack.fccc.edu/PISCES.php). In the final datasets, HIV-1 dataset consisted of 242 non-redundant protein sequences and HIV-2 dataset consisted of 86 non-redundant protein sequences.

**Table 4:** The distributions of sequence identity for HIV-1 proteins and HIV-2 proteins.

Sequence identity	HIV-1 proteins	HIV-2 proteins
≤25%	17	8
≤40%	17	8
≤60%	23	11
≤80%	101	44
≤90%	242	86
≤100%	381	109

### Classifiers

In this study, the increment of diversity (ID)^26^, support vector machine (SVM)^25^, logisitic regression (LR), and multilayer perceptron (MP) were used to classify the HIV-1 proteins and HIV-2 proteins. The C++ software was used to write the ID algorithm, and the SVM, LR and MP algorithms were implemented in the Weka package^69^.

### Protein sample representation

The appropriate parameters were also important for the classifiers. Here, the 20 amino acid compositions, 400 dipeptide compositions, 6 amino acid hydropathy compositions and 36 hydropathy dipeptide compositions were selected as the input parameters of the ID algorithm^44,45^.

### Statistical analysis

In this study, the F-score^70^ was used to quantify the observed difference between the 20 amino acid compositions of the HIV-1 proteins and those of the HIV-2 proteins. The Wilcoxon rank-sum test was carried out to calculate the P-values between the 20 amino acid compositions in the two HIV protein groups. The difference was considered significant if the P-value < 0.05.

### Evaluation of methods

The jackknife test was applied to examine the prediction power of the algorithms. In order to estimate the accuracy of our algorithms, the sensitivity (Sn), specificity (Sp), correlation coefficient (CC) and overall accuracy (Acc) were also calculated^33^.
